# Hormonal Therapy and Cardiovascular Health: Examining the Risk Among Premenopausal Women Diagnosed With Breast Cancer

**DOI:** 10.1016/j.jaccao.2024.10.009

**Published:** 2024-12-17

**Authors:** Farzin Khosrow-Khavar, Laurent Azoulay

**Affiliations:** a Center for Pharmacoepidemiology and Treatment Science, Institute for Health, Healthcare Policy and Aging Research, Rutgers, The State University of New Jersey, New Brunswick, New Jersey, USA; b Department of Biostatistics and Epidemiology, School of Public Health, Rutgers, The State University of New Jersey, Piscataway, New Jersey, USA; c Rutgers Cancer Institute, New Brunswick, New Jersey, USA; d Centre for Clinical Epidemiology, Lady Davis Institute, Jewish General Hospital, Montreal, Quebec, Canada; e Department of Epidemiology, Biostatistics, and Occupational Health, McGill University, Montreal, Quebec, Canada; f Gerald Bronfman Department of Oncology, McGill University, Montreal, Quebec, Canada

**Keywords:** breast cancer, cardiovascular disease, hormonal therapy, premenopausal

**Figure:**
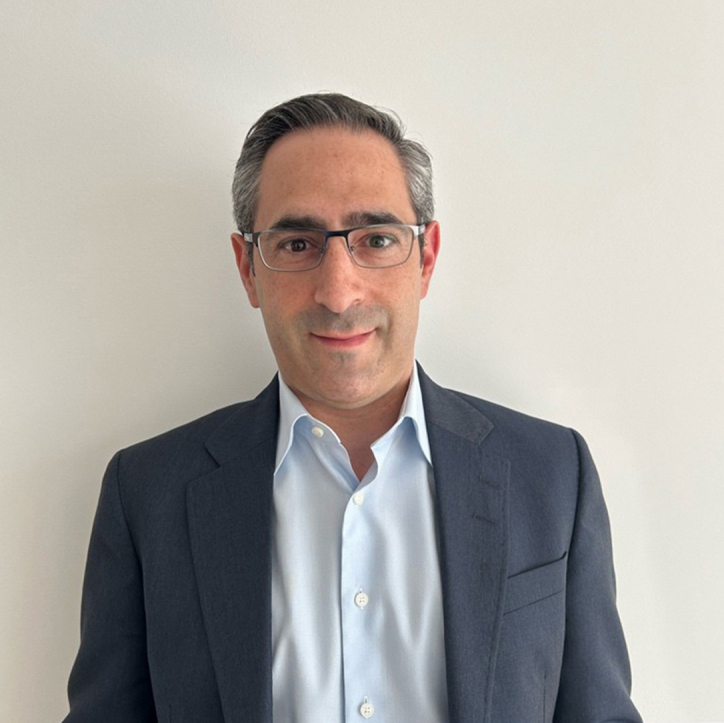

Cardiovascular disease (CVD) is the leading cause of mortality among women diagnosed with breast cancer, posing a significant health risk alongside cancer itself.^1^ Women diagnosed with breast cancer face an increased risk for CVD due to lifestyle-related factors (including smoking, alcohol, sedentary lifestyle, and diet), a burden of comorbidities, and the cardiovascular effects of cancer treatments.^1^

Among patients diagnosed with hormone receptor–positive breast cancer, hormonal therapies, including aromatase inhibitors (AIs) and tamoxifen, are recommended long-term treatments.^2^^,^^3^ However, there has been ongoing debate regarding the comparative risk for CVD with these treatments. Evidence from randomized clinical trials (RCTs) has shown that among postmenopausal women with hormone receptor–positive breast cancer, AIs are associated with an increased risk for CVD compared with tamoxifen.^4^ Among premenopausal women diagnosed with hormone receptor–positive breast cancer, AIs are administered alongside ovarian suppression, either orchiectomy or gonadotropin-releasing hormone agonists.^3^ However, because of limited sample sizes in RCTs, the cardiovascular safety of AIs plus ovarian suppression compared with tamoxifen remains unclear among premenopausal women, a younger group with lower baseline cardiovascular risk.^5^ In the absence of additional RCTs, real-world evidence (RWE) can play a crucial role in understanding the cardiovascular safety of these treatment strategies.

In a study reported in this issue of *JACC: CardioOncology*, Polter et al^6^ conducted the first large RWE study to compare the cardiovascular safety of AIs plus ovarian suppression with tamoxifen in clinical practice. This study used the MarketScan administrative claims databases to identify 47,182 women diagnosed with breast cancer from 2013 and 2020. The study population, with an average age of 44 years, included only those without histories of CVD. Using a propensity score–matched design, 1,164 women initiating AIs and ovarian suppression were matched with 5,058 women initiating tamoxifen. The study controlled for predefined confounders such as demographics, cancer treatments, and comorbidities. The primary cardiovascular endpoint was a composite of myocardial infarction, atrial fibrillation, heart failure hospitalization, stroke, coronary revascularization, and angina. Over a median of 1.6 years, the investigators report a significant increase in the relative hazard of CVD with AIs plus ovarian suppression compared with tamoxifen (HR: 2.20; 95% CI: 1.57-3.09), corresponding to 12 excess cases per 1,000 person-years. An increased relative hazard was also observed for hyperlipidemia (HR: 1.49; 95% CI: 1.00-2.23), corresponding to 7 excess cases per 1,000 person-years. A modest increased relative hazard was also observed for hypertension, although the CI included the null value (HR: 1.19; 95% CI: 0.88-1.60). Similar results were found when CVD was defined more narrowly as a composite of myocardial infarction, atrial fibrillation, heart failure, or ischemic stroke, and the findings were consistent across sensitivity analyses addressing confounding and immortal-time bias.

The increased risk for CVD and hyperlipidemia observed with AIs plus ovarian suppression compared with tamoxifen may be due to the detrimental effects of AIs or ovarian suppression or, alternatively, the cardioprotective properties of tamoxifen.^4^ In postmenopausal women, RCTs comparing tamoxifen with placebo have demonstrated a protective effect on CVD risk (RR: 0.67; 95% CI: 0.45-0.98), while no increased risk has been observed with AIs in RCTs comparing these drugs with placebo or no treatment.^4^ The cardioprotective effects of tamoxifen are likely mediated through its beneficial impact on lipid profiles^7^; tamoxifen has been shown to reduce levels of low-density lipoprotein and total cholesterol by approximately 20% within 1 year of treatment initiation, with effects lasting up to 5 years.^8^, ^9^, ^10^ Other mechanisms, such as anti-inflammatory effects, reductions in C-reactive protein and fibrinogen levels (both strongly associated with CVD), and antioxidant properties that prevent low-density lipoprotein oxidation may also contribute.^10^, ^11^, ^12^, ^13^, ^14^, ^15^ Tamoxifen may also directly improve vascular action by increasing flow-mediated dilation and decreasing carotid intima-media thickness.^16^ Regarding gonadotropin-releasing hormone agonists, most cardiovascular safety data are derived from studies in prostate cancer, in which these agents have generally been associated with an increased risk for CVD.^17^ However, additional evidence is needed among premenopausal women diagnosed with breast cancer, as hormonal dynamics differ significantly between these populations.

Overall, the study by Polter et al^6^ provides valuable evidence on the cardiovascular risks associated with AIs plus ovarian suppression compared with tamoxifen. Nevertheless, several questions remain unanswered. Future studies should assess the long-term differential effects of these treatments on CVD risk, particularly as women transition from premenopausal to postmenopausal status. Additional RWE studies are necessary to evaluate the risk for individual cardiovascular endpoints, each with distinct etiologies and pathophysiologies. Research is also needed to identify high-risk subgroups, including patients with histories of CVD, high comorbidity burden, or significant cardiovascular risk factors. In future studies, careful consideration of study design challenges, such as immortal-time bias and potential confounding by indication, will be essential.

Ultimately, the decision between AIs plus ovarian suppression vs tamoxifen should consider both efficacy and a comprehensive safety profile. A recent individual patient data meta-analysis indicated that AIs combined with ovarian suppression were associated with a 21% reduction in breast cancer recurrence (RR: 0.79; 95% CI: 0.69-0.90) but with no differences in breast cancer mortality (RR: 1.01; 95% CI: 0.82-1.24).^5^ However, additional follow-up is needed to assess differential effects on long-term outcomes. Overall, the current body of evidence suggests that women undergoing hormonal therapy for breast cancer could benefit from more intensive cardiovascular monitoring to improve patient management and cardiovascular outcomes.^17^

## Funding Support and Author Disclosures

Dr Khosrow-Khavar is supported by a career development award from the American Heart Association (24CDA1259477). Dr Azoulay has received speaking and consulting fees from Roche and Pfizer for unrelated work.
